# Effects of Low Frequency Prefrontal Repetitive Transcranial Magnetic Stimulation on the N2 Amplitude in a GoNogo Task

**DOI:** 10.1371/journal.pone.0067136

**Published:** 2013-06-27

**Authors:** Nicola Grossheinrich, Maren Reinl, Oliver Pogarell, Susanne Karch, Christoph Mulert, Monika Brueckl, Kristina Hennig-Fast, Anne Rau, Maria Epple, Ariane Hornig, Frank Padberg

**Affiliations:** 1 Department of Psychiatry, Ludwig-Maximilians University, Munich, Germany; 2 Child Neuropsychology Section, Department of Child and Adolescent Psychiatry and Psychotherapy, University Hospital of the RWTH Aachen, Aachen, Germany; 3 Werner Reichardt Centre of Integrative Neuroscience (CIN), Tuebingen, Germany; 4 University Medical Center Hamburg-Eppendorf, Department of Psychiatry and Psychotherapy, Psychiatry Neuroimaging Branch (PNB), Hamburg, Germany; 5 Department of Child and Adolescent Psychiatry, Psychosomatic and Psychotherapy, Ludwig-Maximilian University, Munich, Germany; 6 Department of Psychology, Eberhard-Karl University, Tuebingen, Germany; University of Regensburg, Germany

## Abstract

During the last decade, repetitive transcranial magnetic stimulation (rTMS) of the prefrontal cortex has become established as a treatment for various mental diseases. The rational of prefrontal stimulation has been adapted from the mode of action known from rTMS using motor-evoked potentials though little is known about the precise effect of rTMS at prefrontal sites. The objective of the current study is to investigate the inhibitory effect of prefrontal 1 Hz rTMS by stimulating the generators of event-related potentials (ERP) which are located in the prefrontal cortex. Thus, 1 Hz rTMS was applied offline over the left dorsolateral prefrontal cortex (DLPFC) and the medial prefrontal cortex (MPFC) in 18 healthy subjects who subsequently underwent a GoNogo task. Both active conditions were compared to sham rTMS within a randomized and counterbalanced cross-over design in one day. ERPs were recorded during task performance and the N2 and the P3 were analysed. After 1 Hz rTMS of the left DLPFC (but not of the MPFC), an inhibitory effect on the N2 amplitude was observed, which was related to inhibitory control. In contrast, after 1 Hz rTMS of the MPFC (but not at the left DLPFC) a trend towards an increased P3 amplitude was found. There was no significant modulation of latencies and behavioural data. The results argue in favour of an inhibitory effect of 1 Hz rTMS on N2 amplitudes in a GoNogo task. Our findings suggest that rTMS may mildly modulate prefrontally generated ERP immediately after stimulation, even where behavioural effects are not measurable. Thus, combined rTMS-ERP approaches need to be further established in order to serve as paradigms in experimental neuroscience and clinical research.

## Introduction

During the last decade, prefrontal repetitive transcranial magnetic stimulation (rTMS) has become increasingly established as a treatment for various mental diseases. For this purpose the rationale of stimulation has been adapted from the mode of action known from motor-evoked potentials (MEP). For example, in major depression a hemispheric asymmetry is assumed due to lower activity in the left compared to the right hemisphere [1]. Hence major depression is treated by left excitatory [2] or right inhibitory prefrontal rTMS [3]. Analogue to the inhibiting motor effect [4], 1 Hz rTMS was applied to the prefrontal cortex of patients afflicted by mental diseases characterized by hyperexcitability such as the Tourette syndrome [5], [6], post-traumatic stress disorder [7] or obsessive compulsive disorder [8].

Some imaging studies of prefrontal 1 Hz rTMS have been conducted showing decreasing [9] or increasing metabolism [10]. Likely, these contradictory results are attributed to indirect transsynaptical rTMS effects combined with brain activity measurements which are mediated by metabolism. Therefore, neural oscillations which can be directly measured comparable to MEPs of rTMS at motor sites – such as event-related potentials (ERPs) – are a promising tool to estimate the mode of action of prefrontal rTMS.

In neurophysiological research, the N2 and P3 in GoNogo tasks are considered as ERPs associated with inhibitory control [11] – an executive process that is aberrant in some mental diseases [12]. Moreover, the N2 is assumed to be generated in prefrontal cortices, namely the anterior cingulate cortex (ACC, [13]–[15]) and the dorsolateral prefrontal cortex (DLPFC, [16]), whereas the P3 is located in frontocentral [17] but also in parietal regions [18].

Until today, two paradigms had been used to investigate the impact of 1 Hz rTMS on the mechanism of inhibitory control in neurophysiological research [19], [20]. In one study, subjects performed a stop signal task immediately before and after a train of 1 Hz rTMS had been applied to the right and left DLPFC. However, neither a change in ERPs (N2, P3) nor in behavioural measures was detectable [20]. The second study investigated the error-related negativity (ERN, [19]) using a flanker task, which has been hypothesized to be functionally comparable to the N2 component [15], although the dissociation of both has been demonstrated [21], [22]. In this study, an attenuation of the ERN was found after applying 1 Hz rTMS at the medial prefrontal cortex (MPFC) but not after lateral frontal stimulation. Moreover, the authors found enhanced error positivity (Pe) and a decreased rate of corrections [19].

Though research of rTMS on ERPs has been conducted, no study was intended to estimate the mode of action of prefrontal rTMS. Some studies have explored 1 Hz rTMS effects by positron emissions tomography (PET) leading to contradictory results [9], [10].

Here, the mode of action of prefrontal 1 Hz rTMS is investigated in a GoNogo task by stimulating the generators of the N2 component known to be located in the prefrontal cortex. Therefore three different 1 Hz rTMS conditions (active left DLPFC, active MPFC and sham rTMS) are applied offline in a crossover design followed by ERP recording during a GoNogo paradigm. The N2 is analysed in both active conditions in contrast to sham control in the context of the P3 and behavioural data. As it is assumed that one train of prefrontal 1 Hz rTMS would have only a short inhibitory effect [23], an inhibitory influence on the N2 amplitude could be expected immediately after 1 Hz rTMS (t1: 0–15 min) but not in a delayed time frame (t2: 16–30 min). Since it is presumed to modulate the generators of the N2, a differential effect on Go- and Nogo-trials is not expected.

## Materials and Methods

### 1 Ethics Statement

The experiment was conducted in accordance to the Declaration of Helsinki and approved by the ethical review committee of the Ludwig Maximillians University’s medical faculty (project 239–98, Amendment 1). Written informed consent was obtained.

### 2 Participants

Eighteen healthy right-handed volunteers (10 male) aged between 20 and 33 years (M = 24; SD = 3) participated in the study. All subjects were naïve to TMS and were paid for their participation. The subjects were recruited by local announcement. As a first step, a telephone interview was conducted to obtain information about possible neurological or psychiatric diseases of the subjects. Secondly, the subjects were invited to a preliminary investigation where a neuropsychological screening of executive functions was accomplished and the resting motor threshold (RMT) was determined. Only subjects with no history of neurological or psychiatric disorders and a neuropsychological performance at average or above were included.

### 3 GoNogo Task

ERPs were elicited by a visual GoNogo task (1200 stimuli) using 75% Go- and 25% Nogo-trails. Stimuli for Go- and Nogo-trials were circles filled with and without a grid pattern which were presented in the centre of the screen. The order of the stimuli was pseudo-randomized with a variable interstimulus interval (ISI) of 900, 1000, 1100, 1200 or 1300 ms. Participants were instructed to press a button as fast as possible when the Go-stimulus appeared and to withhold their response when the Nogo-stimulus emerged. Stimulus disappeared as soon as the button had been pressed thus subjects could influence the speed of the task. The task was divided into two halves (t1: 0–15 min; t2: 16–30 min) to analyse the immediate and the delayed effect of 1 Hz rTMS. Each half consisted of two blocks. The influence of handedness was controlled by the alteration of the performing hand within t1 and t2 resulting in four possible sequences (right/left – right/left; right/left – left/right; left/right – left/right; left/right – right/left). Each sequence of handedness was held constant within the experiment but was pseudo-randomized and counterbalanced between subjects. Previous to the task, a training session was available presenting an acoustic warning signal when the subject made a mistake (64 stimuli, ISI: 1000 ms).

### 4 rTMS

**Figure 1:**
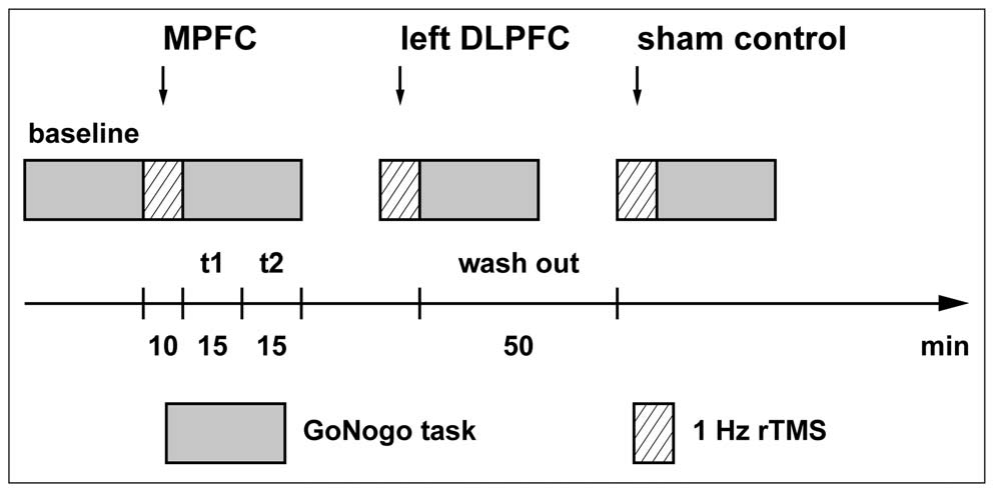
Experimental crossover design counterbalanced for stimulation sites.

The experiment was conducted in a crossover design during a single day [24]. Subjects were seated in a comfortable chair with a distance to the monitor of approximately 1 m (approx. 39 in). After a baseline measurement (including the training session), a train of rTMS was applied at three stimulation sites (MPFC, left DLPFC, sham control) in a fully counterbalanced (six possible sequences performed three times) and randomized order to control for possible carry-over and/or sequence effects (Figure 1). After each rTMS train the subject performed the GoNogo task, which lasted approximately 30 min. Electroencephalogram (EEG) was recorded during task performance at baseline and after TMS application. The interval between stimulation conditions was 50 min to exclude carry-over effects, as the rTMS protocol was expected to induce post-stimulation effects lasting only few minutes [23].

A Magstim Super Rapid Magnetic Stimulator (Magstim Company Ltd, Whitland, UK) with a figure-8-shaped 70 mm coil was used for rTMS. RTM was determined on a separate day prior to the experimental session. Motor evoked potentials (MEPs) were recorded from the abductor pollicis brevis (APB) and defined as the minimum stimulus intensity that produced a liminal motor-evoked potential (>50 µ in at least 50% of 10 trials). Stimulation sites were defined on the basis of the International 10–20 EEG system. The frontopolar region targeting the ACC was stimulated at Fz [25] and left DLPFC was stimulated at the F3 position [26]. For sham rTMS the auditory cortex was chosen corresponding to the T3 electrode intending not to interfere with the visual task. For sham rTMS, the coil was held at an angle of 45° to the scalp to be sure not to exert an effective stimulation. Stimulations were applied at 120% of the individual RMT with a frequency of 1 Hz for 10 minutes (600 impulses). During rTMS, the respective electrode was transiently removed and replaced immediately afterwards.

### 5 EEG-recording

EEG was recorded with 33 Ag/AgCl electrodes referred to Cz (32 channels: Fp1, Fp2, F3, F4, F7, F8, C3, C4, P3, P4, T5, T6, T3, T4, O1, O2, Fz, Cz, Pz, Oz, A1, A2, T1, T2, Fc5, Fc6, Fc1, Fc2, Cp5, Cp6, P9, P10). An EEG-Cap was used with removable electrodes to allow for application of rTMS. Electrodes were positioned according to the International 10–20 system. For the recording of eye movements an additional electrode (EOG) was positioned 1 cm lateral to the left eye. Electrode skin impedance was less than 5 kΩ at the beginning of the session. Data were collected with a sampling rate of 250 Hz and an analogous band pass filter (0.16 - 200 Hz).

### 6 Sensation Seeking Personality Traits

To further investigate the relationship between ERP (N2, P3) and inhibitory control the Sensation Seeking Scale (SSS-V) was applied, which was developed by Zuckerman [27]. Sensation Seeking is a highly heritable trait [28] and is linked to impulsivity and novelty seeking [29]. The SSS-V consists of 40 items and four subscales: Thrill and Adventure Seeking (TAS), Disinhibition (DIS), Experience Seeking (ES), and Boredom Susceptibility (BS). The present study used a standardized German translation of Zuckerman's SSS-V. The reliability of the German version is reported as acceptable with an alpha coefficient of.82 [30].

### 7 Data analysis

#### 7.1 Event related potentials

The 32 channels were considered off-line in contrast to an average reference. Signals were filtered with a 30 Hz (24db/oct) low pass and a notch filter (50 Hz). Continuous EEG was segmented into 1 second-epochs starting 100 ms before stimulus onset for Go- and Nogo-trials separately. ERPs were only calculated for trials containing correct responses. Trials with artefacts to a criterion of 70 µV maximal amplitude were rejected from further analysis. After baseline correction (−100 ms) the remaining segments were averaged. Only ERPs consisting of 30 trials or more were kept. Frontocentral electrodes of the midline (Fz and Cz) were selected for statistical analysis [31]–[33]. Individual peak amplitudes and latencies were selected and observed between 200 ms to 328 ms for the N2 and between 280 ms to 464 ms for the P3 component regarding the Cz and Fz electrode.

#### 7.2 Statistics

Statistical analysis was conducted for an immediate and delayed time frame to exclude carry-over effects and to ensure the validity of an rTMS effect. As a single train of prefrontal rTMS is assumed to exert only a short effect [23], an influence on behavioural and ERP data is expected immediately after stimulation but not in a delayed time slot. Peak amplitudes and latencies (N2, P3) were examined comparing all conditions (MPFC, left DLPFC, sham control). Four-factorial ANOVAs were conducted including the within-factors ‘stimulation site’ (MPFC, left DLPFC, sham control), ‘electrodes’ (Fz, Cz), ‘trial type’ (Go, Nogo) and ‘time frame’ (t1: 1–15 min, t2: 16–30 min). A priori contrasts were calculated for each active condition compared to sham control. One data set (MPFC) was lost; therefore initial analyses were carried out with 17 subjects only.

Mean reaction time and mistakes (omissions and false alarms) were analysed individually for all conditions (MPFC, left DLPFC, sham control). For behavioural data ANOVAs were conducted containing the within factors ’stimulation site’ (MPFC, left DLPFC, sham control) and ‘time frame’ (t1: 1–15 min, t2: 16–30 min).

Post hoc, baseline measurements were used to investigate principle characteristics concerning the N2, P3 and the sensation seeking personality trait. Analyses of variance (ANOVA) were conducted for the within-factors ‘electrode’ (Fz, Cz), ‘trial type’ (Go, Nogo) and the between-factor ‘disinhibition’ (SSS-V, DIS, median split: high, low). Correlations between dependent variables (mean reaction time, mistakes, N2, P3) and sensation seeking were performed for the baseline using Pearson coefficients. For calculating correlations, mean amplitudes were averaged across electrode positions [34]. Hierarchical regression analysis was carried out including significant correlations, gender and age. Significance level was set at p<.05. Trends were set at p<.1 and were reported for exploratory analyses (P3, behavioural data).

## Results

### 1 Behavioural Data

ANOVA including the within-factors ‘stimulation site’ and ‘time frame’ showed a slower mean reaction time for the second in contrast to the first time frame (F [1], [17] = 7.16, p = .02, see Table S1). Neither mean reaction time nor mistakes (false alarms and omissions) were influenced by prefrontal 1 Hz rTMS (Table S1, supporting information).

### 2 Event-related Potentials

#### 2.1 Experiment – N2

**Figure 2:**
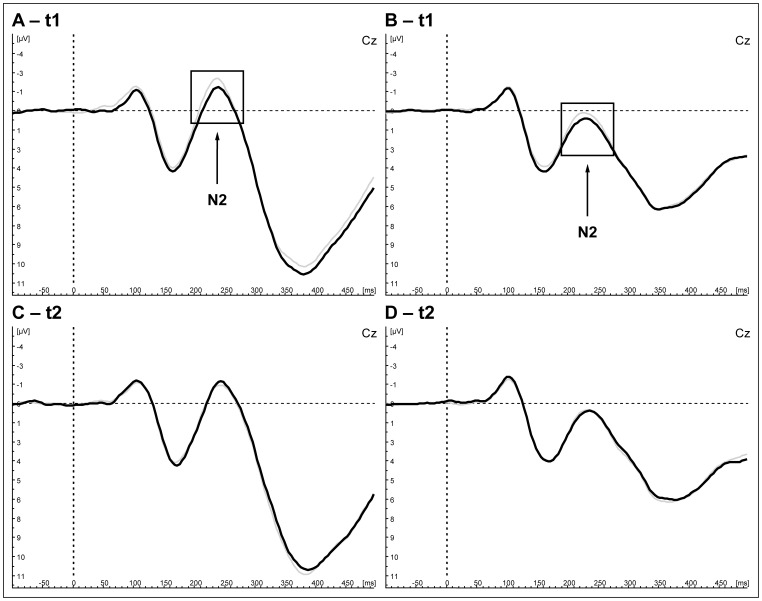
N2 difference after stimulation overthe left DLPFC (solid lines) compared to sham control (grey lines). Illustrated are grand averages for Nogo (A, C) and Go trials (B, D) immediately after stimulation (t1; A, B) and delayed (t2; C, D).

Four-factorial ANOVA (‘stimulation site (3)’: MPFC, left DLPFC, sham control x ‘trial (2)’: Go, Nogo x ‘electrodes (2)’: Fz, Cz x ‘time frame (2)’: t1: 1–15 min, t2: 16–30 min) yielded a significant interaction between ‘stimulation site’, ‘electrodes’ and ‘time frame’ (F [1]; [16] = 3.49, p = .04, partial η^2^ = .18). Contrasts compared to sham control yielded a significant effect for left DLPFC rTMS (F [1]; [16] = 4.77, p = .04, partial η^2^ = .23) but not for MPFC rTMS (F [1]; [16] = 3.39, p = .08) which shows a statistical trend regarding the reported interaction effect. Three-factorial ANOVA (‘stimulation site (2)’: left DLPFC, sham control x ‘electrodes (2)’: Fz, Cz x ‘trial type (2)’: Go, Nogo) including all subjects (N = 18) was conducted for each time frame separately to investigate the reported interaction effect further on. The ‘stimulation site’ x ‘electrodes’ interaction was observed in the first time-frame (t1: 1–15 min) of left DLPFC rTMS (F [1]; [17] = 8.19, p = .01, partial η^2^ = .33, Table 1) and is illustrated in the grand averages (Figure 2, A: Nogo, B: Go) as a diminishment of the N2-amplitude at Cz in contrast to the sham control condition. Post hoc t-test revealed significant decreased N2-amplitudes at Cz in contrast to Fz (T [17] = 2.86, p = .01) and a small to moderate effect size (Cohen’s d = 0.27). According to our hypothesis, this interaction was absent in the delayed time frame (t2: 16–30 min, Figure 2, C: Nogo, D: Go) following rTMS of left DLPFC (F [1]; [17] = 0.001, p = .98, Table 1). The reported effects did not alter when only the restricted number (one drop out of the MPFC rTMS condition) of subjects (N = 17) was included in the analysis.

**Table 1:** ANOVA of the N2 amplitude over the left DLPFC.

within factor	time frame t1		time frame t2	
N = 18, df = 1	F	p	F	p
electrodes (Fz, Cz)	14.43	<.01	13.14	<.01
trial type (Go, Nogo)	15.64	<.01	12.75	<.01
stimulation site (left DLPFC, sham control)	0.12	.74	0.18	.67
stimulation site × trial type	0.04	.84	1.88	.19
stimulation site × electrodes	8.19	.01	0.001	.98
trial type × electrodes	13.48	<.01	12.83	<.01
trial type × electrodes × stimulation site	0.001	.98	0.002	.97

**Figure 3:**
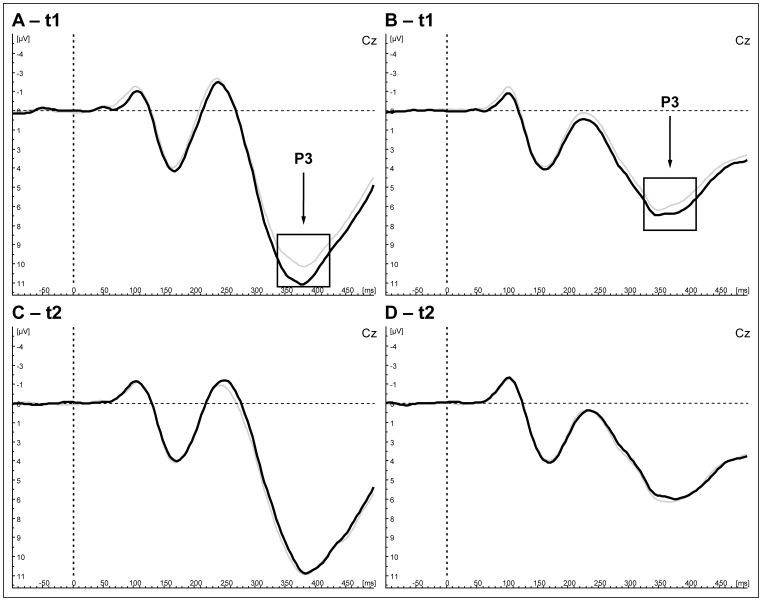
P3 difference after stimulation overthe mPFC (solid lines) and sham control (grey lines). Illustrated are grand averages for Nogo (A, C) and Go trials (B, D) immediately after stimulation (t1; A, B) and delayed (t2; C, D; N = 17).

The statistical trend concerning the contrast between MPFC rTMS and sham control could not be detected in further three-factorial analysis (all ps concerning the ‘stimulation site’ >.1) and was not visible in the grand average (see Figure 3, A: Go, B: Nogo).

Alongside, four-factorial ANOVA yielded an interaction of ‘electrodes’ and ‘trial type’ (F [1]; [16] = 11.63, p<.01, partial η^2^ = .42) with a larger discrepancy for the Go- and Nogo-trials at the Cz electrode in comparison to the Fz electrode. Main effects for ‘trial type’ (F [1]; [16] = 14.64, p<.01) and ‘electrodes’ (F [1]; [16] = 17.03, p<.01) were found with larger amplitudes for the Nogo trails and the Fz electrode. The main effect of ‘electrodes’, ‘trial type’ and the interaction of both remained constant independent of the time frame (Table 1).

Moreover a main effect of the ‘time frame’ was found with decreasing N2 amplitudes for the second compared to the first time frame. No other effects were found in the four-factorial analysis (all p>.1). Latencies were not affected by 1 Hz rTMS.

#### 2.2 Experiment – P3

Four-factorial ANOVA (‘stimulation site (3)’: MPFC, left DLPFC, sham control × ‘trial (2)’: Go, Nogo x ‘electrodes (2)’: Fz, Cz x ‘time frame (2)’: t1: 1–15 min, t2: 16–30 min) showed a statistical trend for ‘stimulation site’, ‘electrodes’ and ‘time frame’ (F [1]; [16] = 3.02, p = .06, partial η^2^ = .16). Contrasts yielded a significant effect for MPFC rTMS (F [1]; [16] = 5.48, p = .03, partial η^2^ = .26) but not for left DLPFC rTMS (F [1]; [16] = 1.01, p = .33) compared to sham stimulation regarding the reported interaction. Three-factorial ANOVA (‘stimulation site (2)’: MPFC, sham control) x ‘electrodes (2)’: (Fz, Cz’) x ‘trial type (2)’: (Go, Nogo) was conducted for each time frame separately to investigate the reported interaction effect further on. The trend was based on the ‘stimulation site’ x ‘electrode’ interaction (F [1]; [16] = 4.76, p = .04, partial η^2^ = .23, Table 2) immediately after MPFC rTMS compared to sham control and was visible as an enhanced P3 amplitude at the Cz electrode in the grand average (Figure 3, A: Nogo, B: Go). Post hoc t-test revealed significant increased P3 amplitudes at Cz compared to Fz (T (16) = 5.2, p<.01) and a small estimated effect size (Cohen’s d = 0.17). The reported trend was not detected in the delayed time frame (F [1]; [16] = 0.28, p = 0.60; Table 2, Figure 3, C: Nogo, D: Go).

**Table 2:** ANOVA of the P3 amplitude over the MPFC.

within factor	time frame t1		time frame t2	
N = 17, df = 1	F	p	F	p
electrodes (Fz, Cz)	30.19	<.01	29.97	<.01
trial type (Go, Nogo)	66.09	<.01	70.19	<.01
stimulation site (left DLPFC, sham control)	0.60	.45	0.00	.96
stimulation site × trial type	0.00	.99	1.21	.28
stimulation site × electrodes	4.76	.04	0.28	.60
trial type × electrodes	5.70	.03	7.14	.02
trial type × electrodes × stimulation site	1.45	.25	0.40	.54

Alongside, an interaction of ‘electrodes’ and ‘trial type’ with a larger discrepancy of Go- and Nogo-trials at Cz electrode in comparison to the Fz electrode was observed (F [1]; [16] = 7.57, p = 0.01, partial η^2^ = .32). Main effects for ‘trial type’ (F [1]; [16] = 77.4, p<.01, partial η^2^ = .83) and ‘electrodes’ (F [1]; [16] = 30.45, p<.01, partial η^2^ = .66) were found with larger amplitudes for the Nogo trails and the Cz electrode. The main effect of ‘electrodes’, ‘trial type’ and the interaction of both remained constant independent of the time frame (Table 1). No other effects were found in the four-factorial analyses (all p>.1). No stimulation effects on latencies were observed (p>.1).

#### 2.3 N2 in relation to disinhibition

At baseline, the mean reaction time of the GoNogo task was 306.89 ms (SD = 57.69 ms) with a mean false alarm rate of 33.89% (SD = 29.16%) and a mean omission rate of 0.28% (SD = 0.40%). The mean peak amplitude of N2 emerged at 242.14 ms (SD = 18.42), i.e. 64.89 ms prior to the mean reaction time.

**Figure 4:**
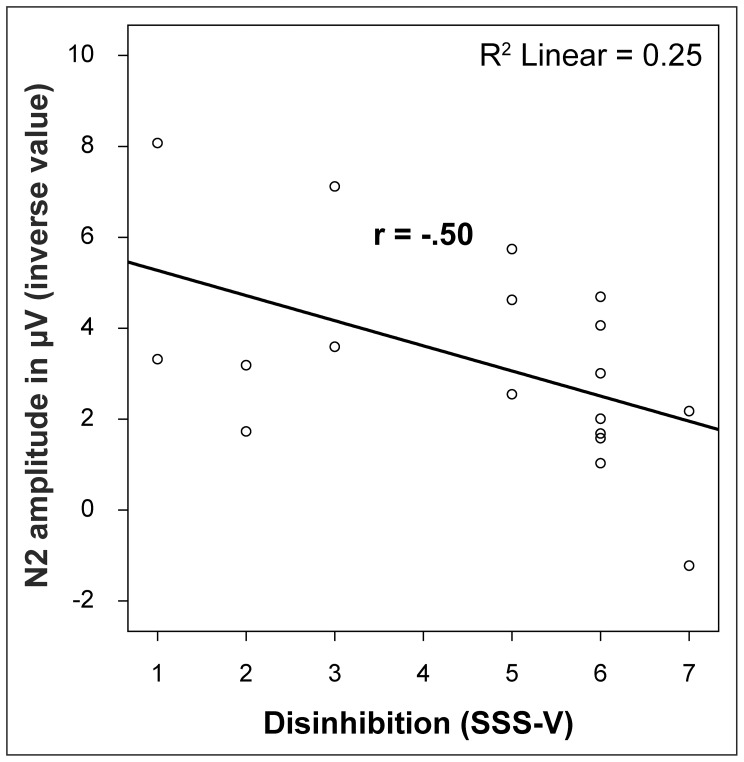
Negative relationship between the Disinhibition subscale of the Sensation Seeking Scale and the N2 amplitude.

As there were no significant effects of rTMS on behavioural data, changes in the N2 amplitude could not be related to behavioural measures. Thus, the N2 was further analysed at baseline in relation to trait measures of sensation seeking (SSS-V) as a highly heritable trait [28] which is linked to impulsivity and novelty seeking [29]. N2 peak amplitudes (inverse value) were negatively associated with SSS-V (total score: r (18) = −.48, p = .045), especially with the subscale DIS (r (18) = −.50, p = .03) and ES (r (18) = −.48; p = .04). In a hierarchical regression analysis the DIS subscale remained as significant predictor and accounted 25% for the variance demonstrating reduced amplitudes for high disinhibited subjects (Figure 4). No influence of gender or age was observed.

An ANOVA of the N2 amplitude including the within factors ‘electrode’ (Fz, Cz), ‘trial type’ (Go, Nogo) and the between factor ‘disinhibition’ (SSS-V, DIS, median split: high, low) yielded a significant effect of disinhibition (F [1]; [16] = 6.42, p = .02) with low amplitudes for high and high amplitudes for low disinhibited individuals. No interaction concerning ‘disinhibition’ was observed (all ps >.1).

Mean peak P3 amplitudes occurred at 377.64 ms (SD = 20.73), i.e. 70.75 ms after mean reaction time and was not associated with total SSS-V scores or scores of their subscales. The between factor ‘DIS’ did not reach significance (F [1]; [16] = 0.96, p = 0.34) in an ANOVA of the P3 amplitude with the within-factors ‘electrode’ (Fz, Cz), ‘trial type’ (Go, Nogo) and the between factor ‘disinhibition’ (SSS-V, DIS, median split: high, low).

## Discussion

The main result of the study is a diminished N2 amplitude immediately after 1 Hz rTMS (10 min, 120% RMT) at left DLPFC and a trend for an increased P3 amplitude immediately after 1 Hz rTMS at MPFC. As hypothesized, both effects were absent in a later time frame. The observation that all other reported effects of the N2 and P3 amplitude remained over the whole 30 minute recording period (Table 1 and Table 2) supports the conclusion that the reported effects are genuine rTMS effects.

### 1 Effects on the N2 Amplitude

The objective of the experiment was to investigate the role of the putative generators of the N2 at the left DLPFC and the MPFC for inhibitory control in a GoNogo paradigm [13], [16]. For this purpose we used 1 Hz rTMS which is known to exert inhibitory effects on cortical excitability [4]. A differential effect on Go- or Nogo-trials was not expected given that both amplitudes were modulated to the same amount.

In contrast to the attenuation of the ERN reported by Rollnick et al. [19], no rTMS effect on the N2 amplitude was observed when stimulating the midline. One explanation could be the dissociation of both ERPs as suggested by a few authors [21], [22], who found an attenuation of the ERN despite an intact N2.

Moreover, an attenuated N2 amplitude immediately after 1 Hz rTMS of the left DLPFC was detected, which is in line with the inhibitory effect of 1 Hz rTMS. This observation could be explained by a successful direct stimulation of the left DLPFC (as a generator of the N2 amplitude [16]) or by a transsynaptical rTMS effect possible through the strong interconnections between DLPFC and ACC [35]–[37]. Surprisingly, this attenuation was not accompanied by a modulation of the P3 wave.

The enhancement by trend of the P3 amplitude after 1 Hz rTMS over the MPFC is in favour for an excitatory rTMS modulation and is in line with an increased activity in PET studies [9], [10].

In sum, evidence was found that amplitudes of N2 can be modulated when 1 Hz rTMS is administered to the left DLPFC. Moreover, the enhancement by trend of the P3 amplitude after 1 Hz rTMS over the MPFC implicate that excitatory prefrontal modulations are possible using 1 Hz rTMS.

### 2 Influence on Inhibitory Control

The negative association between DIS (a factor of impulsive control, [29]) and the N2 support the assumption that the N2 amplitude is related to inhibitory control processes. Additionally a prominent ‘electrode × trial type’ effect was observed demonstrating a larger GoNogo difference at Cz than at Fz in any condition for the N2 amplitude. This finding suggests that Cz reflected the GoNogo discrepancy in this experiment the best. Moreover, in line with our hypothesis, an inhibitory effect of 1 Hz rTMS was found which was only visible at Cz. Interestingly, a recent study described the Cz electrode as the best to measure conflict monitoring and response inhibition [38].

The relationship of the N2 amplitude with the personality trait DIS and ES of the SSS-V may also support that 1 Hz rTMS influenced a neurophysiological component which is related to processes of impulsive control [29]. Moreover, sensation seeking is known as a heritable trait [28] and sensation seeking as measured by the SSS-V is well investigated in terms of a neurobiological background. For example, the subscale DIS was associated with low cortisol levels [39] and the total score of SSS was related to low salivary cortisol levels [40]. Another investigation discovered a relation between the DIS subscale of the SSS-V and the serotonergic systems in humans. Among other results the authors found that high compared with low disinhibited individuals exhibited low cortisol responses to a serotonergic challenge (ipsapirone) in a game designed to produce aggression [41]. Moreover the ACC glutamate concentration was negatively correlated with the sensation seeking sum score and the ES subscore [42].

In sum, a negative association between N2 amplitude and the DIS and ES subscales of SSS-V was found, which argues in favour for a modulation of neurophysiological parameters linked to highly heritable personality traits and likely to their neurobiological mechanisms.

### 3 Lack of Behavioural Effects

The missing effect of 1 Hz rTMS on behavioural performance is in line with similar studies [19], [20]. No effect was observed on behavioural measures when 1 Hz rTMS was applied to the left and right DLPFC in a stop signal task [20]. Additionally no impact of 1 Hz rTMS on reaction times and errors rates in a flanker task was found though an attenuation of the ERN was reported after 1 Hz rTMS at the MPFC [19]. Therefore the study is in line with previous findings and suggests that ERP measures may be more sensitive to the impact of rTMS than behavioural data.

In addition, the lack of behavioural results reported here is in line with the core assumption of endophenotype research. Gottesman and Gould (2003) introduced the endophenotype concept as “measurable components unseen by the unaided eye along the pathway between disease and distal genotype” [43]. In this experiment, ERP measurements are not observable in contrast to open behaviour without any supporting tool. Hence, the endophenotype approach is one concept which explains why modifications of ERP data without any accompanying behavioural changes are not contradictory. Specifically, the N2 wave is proved to be heritable [44], which is another criteria of the endophenotype concept [43]. Interestingly, the N2 amplitude is assumed as endophenotype in disorders affecting inhibitory control as attention deficit hyperactivity disorder (ADHD) [45], [46].

In sum, modifications of ERP data accompanied by a lack of behavioural effects is not contradictory and in line with the endophenotype concept. This statement is underlined by the discussion of the N2 wave as endophenotype of ADHD.

### 4 Limitations and Conclusions

In the current study the mode of action of prefrontal 1 Hz rTMS was investigated on N2 amplitudes in the context of behavioural performance and the P3 amplitude in a GoNogo task. Both active conditions were compared to sham rTMS within a randomized and counterbalanced cross-over design in one day.

Our study has several limitations. First, the cross-over design comparing two active and one sham rTMS conditions on the same day is definitely not optimal regarding potential carry-over effects between active and sham conditions, but it has the advantage to exclude interday-variability which may make it more difficult to detect small effects as observed in the current study. Though post-stimulation effects of rTMS are regularly very short-lived and the 1 Hz rTMS effect was observed immediately after stimulation and absent in a second time period, carry-over effects cannot be fully excluded. This argument is serious especially for the observed trend of an enhanced P3 amplitude after 1 Hz rTMS of MPFC as the P3 depend on generators within a complex network [47]. Thus in future studies, rTMS sessions on separate days are recommended and crucial to replicate our experimental findings.

Second, rTMS was targeted based on electrode positions of the 10–20 system, but not neuronavigated to a distinct anatomical location. Recently, it has been shown that standard targets and even anatomical targets are not reliable as the individual connectivity of such targets may be highly variable [48]. Thus, one may consider using individually defined target regions based on resting state connectivity in conjunction with neuronavigated rTMS in future studies.

As hypothesized, an inhibiting effect after 1 Hz rTMS of the left DLPFC was observed on the N2 amplitude which may be related to inhibitory control in a GoNogo task. Unexpected a trend for an excitatory effect was found after 1 Hz rTMS at the MPFC on the P3 amplitude, which should be regarded with caution due to the exploratory nature of this finding.

Despite the fact that replication of our findings is needed, this study supports the idea of establishing ERP-based paradigms for prefrontal rTMS which may serve as experimental means for testing rTMS protocols in a similar manner as motor cortex paradigms have been used previously.

## Supporting Information

Table S1 **Mean reaction time and mistakes of a GoNogo task after 1 Hz rTMS over different stimulations sites.** Means and standard deviations for the immediate (t1: 0–15 min) and delayed (t2: 16–30 min) time frame (N = 18), p values of the ANOVA (stimulation site × time frame).
(DOC)

## References

[1] Thibodeau R, Jorgensen RS, Kim S (2006) Depression, anxiety, and resting frontal EEG asymmetry: a meta-analytic review. Journal of abnormal psychology 115: 715–729. 17100529 10.1037/0021-843X.115.4.715

[2] Schutter DJLG (2009) Antidepressant efficacy of high-frequency transcranial magnetic stimulation over the left dorsolateral prefrontal cortex in double-blind sham-controlled designs: a meta-analysis. Psychological medicine 39: 65–75. 18447962 10.1017/S0033291708003462

[3] Klein E, Kreinin I, Chistyakov A, Koren D, Mecz L, et al (1999) Therapeutic efficacy of right prefrontal slow repetitive transcranial magnetic stimulation in major depression: a double-blind controlled study. Archives of general psychiatry 56: 315–320. 10197825 10.1001/archpsyc.56.4.315

[4] Chen R, Classen J, Gerloff C, Celnik P, Wassermann EM, et al (1997) Depression of motor cortex excitability by low-frequency transcranial magnetic stimulation. Neurology 48: 1398–1403. 9153480 10.1212/wnl.48.5.1398

[5] Mantovani A, Lisanby SH, Pieraccini F, Ulivelli M, Castrogiovanni P, et al (2006) Repetitive transcranial magnetic stimulation (rTMS) in the treatment of obsessive-compulsive disorder (OCD) and Tourette’s syndrome (TS). The international journal of neuropsychopharmacology/official scientific journal of the Collegium Internationale Neuropsychopharmacologicum 9: 95–100. 10.1017/S1461145705005729 15982444

[6] Kwon HJ, Lim WS, Lim MH, Lee SJ, Hyun JK, et al (2011) 1-Hz low frequency repetitive transcranial magnetic stimulation in children with Tourette’s syndrome. Neuroscience letters 492: 1–4. 21256925 10.1016/j.neulet.2011.01.007

[7] Watts BV, Landon B, Groft A, Young-Xu Y (2012) A sham controlled study of repetitive transcranial magnetic stimulation for posttraumatic stress disorder. Brain stimulation 5: 38–43. 22264669 10.1016/j.brs.2011.02.002

[8] Prasko J, Pasková B, Záleský R, Novák T, Kopecek M, et al (2006) The effect of repetitive transcranial magnetic stimulation (rTMS) on symptoms in obsessive compulsive disorder. A randomized, double blind, sham controlled study. Neuro endocrinology letters 27: 327–332. 16816829

[9] Speer AM, Willis MW, Herscovitch P, Daube-Witherspoon M, Shelton JR, et al (2003) Intensity-dependent regional cerebral blood flow during 1-Hz repetitive transcranial magnetic stimulation (rTMS) in healthy volunteers studied with H215O positron emission tomography: II. Effects of prefrontal cortex rTMS. Biological psychiatry 54: 826–832. 14550682 10.1016/s0006-3223(03)00324-x

[10] Knoch D, Treyer V, Regard M, Müri RM, Buck A, et al (2006) Lateralized and frequency-dependent effects of prefrontal rTMS on regional cerebral blood flow. NeuroImage 31: 641–648. 16497518 10.1016/j.neuroimage.2005.12.025

[11] Bokura H, Yamaguchi S, Kobayashi S (2001) Electrophysiological correlates for response inhibition in a Go/NoGo task. Clinical neurophysiology: official journal of the International Federation of Clinical Neurophysiology 112: 2224–2232. 11738192 10.1016/s1388-2457(01)00691-5

[12] Lipszyc J, Schachar R (2010) Inhibitory control and psychopathology: a meta-analysis of studies using the stop signal task. Journal of the International Neuropsychological Society: JINS 16: 1064–1076. 20719043 10.1017/S1355617710000895

[13] Bekker EM, Kenemans JL, Verbaten MN (2005) Source analysis of the N2 in a cued Go/NoGo task. Brain research Cognitive brain research 22: 221–231. 15653295 10.1016/j.cogbrainres.2004.08.011

[14] Jonkman LM, Sniedt FLF, Kemner C (2007) Source localization of the Nogo-N2: a developmental study. Clinical neurophysiology: official journal of the International Federation of Clinical Neurophysiology 118: 1069–1077. 17368096 10.1016/j.clinph.2007.01.017

[15] Van Veen V, Carter C (2002) The anterior cingulate as a conflict monitor: fMRI and ERP studies. Physiology & Behavior 77: 477–482. 12526986 10.1016/s0031-9384(02)00930-7

[16] Lavric A, Pizzagalli DA, Forstmeier S (2004) When “go” and “nogo” are equally frequent: ERP components and cortical tomography. The European journal of neuroscience 20: 2483–2488. 15525290 10.1111/j.1460-9568.2004.03683.x

[17] Fallgatter AJ, Bartsch AJ, Zielasek J, Herrmann MJ (2003) Brain electrical dysfunction of the anterior cingulate in schizophrenic patients. Psychiatry Research: Neuroimaging 124: 37–48. 14511794 10.1016/s0925-4927(03)00072-6

[18] Brass M, Ullsperger M, Knoesche TR, von Cramon DY, Phillips NA (2005) Who comes first? The role of the prefrontal and parietal cortex in cognitive control. Journal of cognitive neuroscience 17: 1367–1375. 16197690 10.1162/0898929054985400

[19] Rollnik JD, Schröder C, Rodríguez-Fornells A, Kurzbuch AR, Däuper J, et al (2004) Functional lesions and human action monitoring: combining repetitive transcranial magnetic stimulation and event-related brain potentials. Clinical neurophysiology: official journal of the International Federation of Clinical Neurophysiology 115: 145–153. 14706482 10.1016/j.clinph.2003.05.001

[20] Upton DJ, Cooper NR, Laycock R, Croft RJ, Fitzgerald PB (2010) A combined rTMS and ERP investigation of dorsolateral prefrontal cortex involvement in response inhibition. Clinical EEG and neuroscience: official journal of the EEG and Clinical Neuroscience Society 41: 127–131. 10.1177/155005941004100304 20722345

[21] Ridderinkhof KR, de Vlugt Y, Bramlage A, Spaan M, Elton M, et al (2002) Alcohol consumption impairs detection of performance errors in mediofrontal cortex. Science 298: 2209–2211. 12424384 10.1126/science.1076929

[22] Swick D, Turken AU (2002) Dissociation between conflict detection and error monitoring in the human anterior cingulate cortex. Proceedings of the National Academy of Sciences of the United States of America 99: 16354–16359. 12456882 10.1073/pnas.252521499 PMC138615

[23] Nyffeler T, Wurtz P, Lüscher H-R, Hess CW, Senn W, et al (2006) Repetitive TMS over the human oculomotor cortex: comparison of 1-Hz and theta burst stimulation. Neuroscience letters 409: 57–60. 17049743 10.1016/j.neulet.2006.09.011

[24] Grossheinrich N, Rau A, Pogarell O, Hennig-Fast K, Reinl M, et al (2009) Theta burst stimulation of the prefrontal cortex: safety and impact on cognition, mood, and resting electroencephalogram. Biological psychiatry 65: 778–784. 19070834 10.1016/j.biopsych.2008.10.029

[25] Kanda M, Mima T, Oga T, Matsuhashi M, Toma K, et al (2003) Transcranial magnetic stimulation (TMS) of the sensorimotor cortex and medial frontal cortex modifies human pain perception. Clinical neurophysiology: official journal of the International Federation of Clinical Neurophysiology 114: 860–866. 12738431 10.1016/s1388-2457(03)00034-8

[26] Herwig U, Satrapi P, Schönfeldt-Lecuona C (2003) Using the international 10–20 EEG system for positioning of transcranial magnetic stimulation. Brain Topography 16: 95–99. 14977202 10.1023/b:brat.0000006333.93597.9d

[27] Zuckerman M (2007) The sensation seeking scale V (SSS-V): Still reliable and valid. Personality and Individual Differences 43: 1303–1305.

[28] Stoel RD, De Geus EJC, Boomsma DI (2006) Genetic analysis of sensation seeking with an extended twin design. Behavior genetics 36: 229–237. 16550452 10.1007/s10519-005-9028-5

[29] Roberti JW (2004) A review of behavioral and biological correlates of sensation seeking. Journal of Research in Personality 38: 256–279.

[30] Beauducel A, Strobel A, Brocke B (2003) Psychometrische Eigenschaften und Normen einer deutschsprachigen Fassung der Sensation Seeking-Skalen, Form V. Diagnostica. 49: 61–72.

[31] Falkenstein M, Hoormann J, Hohnsbein J (1999) ERP components in Go/Nogo tasks and their relation to inhibition. Acta psychologica 101: 267–291. 10344188 10.1016/s0001-6918(99)00008-6

[32] Karch S, Feuerecker R, Leicht G, Meindl T, Hantschk I, et al (2010) Separating distinct aspects of the voluntary selection between response alternatives: N2- and P3-related BOLD responses. NeuroImage 51: 356–364. 20171291 10.1016/j.neuroimage.2010.02.028

[33] Sehlmeyer C, Konrad C, Zwitserlood P, Arolt V, Falkenstein M, et al (2010) ERP indices for response inhibition are related to anxiety-related personality traits. Neuropsychologia 48: 2488–2495. 20434466 10.1016/j.neuropsychologia.2010.04.022

[34] Beste C, Willemssen R, Saft C, Falkenstein M (2010) Response inhibition subprocesses and dopaminergic pathways: basal ganglia disease effects. Neuropsychologia 48: 366–373. 19782093 10.1016/j.neuropsychologia.2009.09.023

[35] Koski L, Paus T (2000) Functional connectivity of the anterior cingulate cortex within the human frontal lobe: a brain-mapping meta-analysis. Experimental Brain Research 133: 55–65. 10933210 10.1007/s002210000400

[36] Mayberg HS, Lozano AM, Voon V, McNeely HE, Seminowicz D, et al (2005) Deep brain stimulation for treatment-resistant depression. Neuron 45: 651–660. 15748841 10.1016/j.neuron.2005.02.014

[37] Paus T, Castro-Alamancos MA, Petrides M (2001) Cortico-cortical connectivity of the human mid-dorsolateral frontal cortex and its modulation by repetitive transcranial magnetic stimulation. The European journal of neuroscience 14: 1405–1411. 11703468 10.1046/j.0953-816x.2001.01757.x

[38] Kropotov JD, Ponomarev VA, Hollup S, Mueller A (2011) Dissociating action inhibition, conflict monitoring and sensory mismatch into independent components of event related potentials in GO/NOGO task. NeuroImage 57: 565–575. 21571079 10.1016/j.neuroimage.2011.04.060

[39] Ballenger JC, Post RM, Jimerson DC, Lake CR, Murphy D, et al (1983) Biochemical correlates of personality traits in normals: An exploratory study. Personality and Individual Differences 4: 615–625.

[40] Shabani S, Dehghani M, Hedayati M, Rezaei O (2011) Relationship of serum serotonin and salivary cortisol with sensation seeking. International journal of psychophysiology: official journal of the International Organization of Psychophysiology 81: 225–229. 21854814 10.1016/j.ijpsycho.2011.06.015

[41] Netter P, Hennig J, Roed IS (1996) Serotonin and dopamine as mediators of sensation seeking behavior. Neuropsychobiology 34 155–165: 46. 10.1159/000119318 8916073

[42] Gallinat J, Kunz D, Lang UE, Neu P, Kassim N, et al (2007) Association between cerebral glutamate and human behaviour: the sensation seeking personality trait. NeuroImage 34: 671–678. 17123835 10.1016/j.neuroimage.2006.10.004

[43] Gottesman II, Gould TD (2003) The endophenotype concept in psychiatry: etymology and strategic intentions. The American journal of psychiatry 160: 636–645. 12668349 10.1176/appi.ajp.160.4.636

[44] Anokhin AP, Heath AC, Myers E (2004) Genetics, prefrontal cortex, and cognitive control: a twin study of event-related brain potentials in a response inhibition task. Neuroscience letters 368: 314–318. 15364418 10.1016/j.neulet.2004.07.036

[45] Albrecht B, Brandeis D, Uebel H, Heinrich H, Mueller UC, et al (2008) Action monitoring in boys with attention-deficit/hyperactivity disorder, their nonaffected siblings, and normal control subjects: evidence for an endophenotype. Biological psychiatry 64: 615–625. 18339358 10.1016/j.biopsych.2007.12.016 PMC2580803

[46] Albrecht B, Brandeis D, Uebel H, Heinrich H, Heise A, et al (2010) Action monitoring in children with or without a family history of ADHD–effects of gender on an endophenotype parameter. Neuropsychologia 48: 1171–1177. 20026087 10.1016/j.neuropsychologia.2009.12.018 PMC2878640

[47] Mulert C, Pogarell O, Juckel G, Rujescu D, Giegling I, et al (2004) The neural basis of the P300 potential. Focus on the time-course of the underlying cortical generators. European Archives of Psychiatry and Clinical Neuroscience 254: 190–198. 15205974 10.1007/s00406-004-0469-2

[48] Fox MD, Buckner RL, White MP, Greicius MD, Pascual-Leone A (2012) Efficacy of transcranial magnetic stimulation targets for depression is related to intrinsic functional connectivity with the subgenual cingulate. Biological Psychiatry 72: 595–603. 22658708 10.1016/j.biopsych.2012.04.028 PMC4120275

