# Supplementary material for: Effects of Low Frequency Prefrontal Repetitive Transcranial Magnetic Stimulation on the N2 Amplitude in a GoNogo Task
Source: PLoS One. 2013 Jun 27;8(6):e67136. doi: 10.1371/journal.pone.0067136 (PMC3694966; doi:10.1371/journal.pone.0067136)
Supplement: Table S1 — Mean reaction time and mistakes of a GoNogo task after 1 Hz rTMS over different stimulations sites. Means and standard deviations for the immediate (t1: 0–15 min) and delayed (t2: 16–30 min) time frame (N = 18), p values of the ANOVA (stimulation site × time frame). (DOC) [file pone.0067136.s001.doc]

Table S1:

Mean reaction time and mistakes of a GoNogo task after 1 Hz rTMS at different stimulations sites. Means and standard deviations for the immediate (t1: 0-15 min) and delayed (t2: 16-30 min) time frame (N = 18), p values of ANOVA (stimulation site x time frame).

| **time frame** |  | **stimulation site** | | |  |
| --- | --- | --- | --- | --- | --- |
|  |  | **left DLPFC** | **MPFC** | **sham control** | ***p* value** |
| **t1** | **RT (ms)** | 307.72 + 47.43 | 301.97 + 34.23 | 304.99 + 36.77 | .40 |
| **t2** | **RT (ms)** | 316.30 + 46.97 | 313.23 + 40.13 | 307.02 + 29.80 |  |
|  | ***p* value** | .02 | | | .10 |
| **t1** | **No. of mist.** | 34.67 + 24.64 | 34.00 + 27.85 | 35.00 + 23.73 | .94 |
| **t2** | **No. of mist.** | 37.00 + 26.98 | 35.83 + 23.88 | 34.56 + 23.08 |  |
|  | ***p*-value** | .51 | | | .64 |

RT = reaction time, No. of mist. = number of mistakes
